# Social virtual reality helps to reduce feelings of loneliness and social anxiety during the Covid-19 pandemic

**DOI:** 10.1038/s41598-023-46494-1

**Published:** 2023-11-07

**Authors:** Keith Kenyon, Vitalia Kinakh, Jacqui Harrison

**Affiliations:** 1 School of Psychology, Faculty of Health and Wellbeing, University of Bolton, Deane Road, Bolton, BL3 5AB UK; 2 Faculty of Biology, Medicine and Health, The University of Manchester, Coupland Building 3, Oxford Road, Manchester, M13 9PL UK

**Keywords:** Quality of life, Human behaviour

## Abstract

Evidence shows that the Covid-19 pandemic caused increased loneliness, anxiety and greater social isolation due to social distancing policies. Virtual reality (VR) provides users with an easy way to become engaged in social activities without leaving the house. This study focused on adults, who were socialising in Altspace VR, a social VR platform, during the Covid-19 pandemic and it explored whether social VR could alleviate feelings of loneliness and social anxiety. A mixed-methods research design was applied. Participants (n = 74), aged 18–75, completed a questionnaire inside the social VR platform to measure levels of loneliness (UCLA 20-item scale) and social anxiety (17-item SPIN scale) in the social VR platform (online condition) and real world (offline condition). Subsequently, a focus group (n = 9) was conducted to gather insights into how and why participants were using the social VR platform. Findings from the questionnaire revealed significantly lower levels of loneliness and social anxiety when in the social VR platform. Lower levels of loneliness and social anxiety were also associated with participants who socialised with a regular group of friends. In addition, findings from the focus group suggested that being part of an online group facilitates stronger feelings of belonging. Social VR can be used as a valuable intervention to reduce feelings of loneliness and social anxiety. Future studies should continue to establish whether social VR can help to encourage group formation and provide people with enhanced social opportunities beyond the COVID-19 pandemic.

## Introduction

On the 11th March 2020 the World Health Organisation declared the rapidly spreading Corona virus outbreak a pandemic^1^ and world governments began to impose enforced social isolation rules. Throughout 2020/2021 the majority of countries imposed lengthy periods of lockdown. The first UK lockdown lasted almost 4 months and during this time only essential travel was permitted and interaction with others from outside the direct household was forbidden^2^. The lock-down caused disruption to daily routines, social activities, education and work. Social distancing measures led to a collapse in social contact. When people experience a reduction in social contact or when the quality of interaction with others is diminished, they can suffer feelings of loneliness. Nearly 7.5 million adults experienced "lockdown loneliness," which is the equivalent to around 14% of the population.^3^ Additionally, the percentage of the UK population reporting loneliness increased from 10% in March 2020 to 26% in February 2021^4^.

### Social isolation and loneliness

Social isolation and loneliness are different. Social isolation is commonly defined as “the state in which the individual or group expresses a need or desire for contact with others but is unable to make that contact”^5^^, p. 731^. Social isolation can occur due to quarantine or physical separation. Due to quarantine measures enforced during lockdown, people faced involuntary social isolation or at least a reduction in their social interactions to the point that their social network was quantitatively diminished^6^. Loneliness is a subjective experience that arises when a person feels that they are isolated and deprived of companionship, lack a sense of belonging, or that their social interactions with others are diminished in either quantity or quality^7^.

### Social isolation, loneliness and detrimental implications for physical and mental health

The rise of loneliness during lockdown also increased the prevalence of anxiety^3^ and such health problems as depressive symptoms and insomnia, reconfirming findings from earlier research^8^ that explored the relationship between social isolation and loneliness and the effect it has on our physical and mental health. Loneliness can lead to stress and high blood pressure, a sedentary or less active lifestyle, and a reduction in cognitive function^9–11^. Loneliness can also lead to less healthy behaviours e.g. an increase in alcohol consumption and smoking^12^, a poor diet^13^ and poor sleeping patterns^14^. Loneliness has been found to have an impact on a person’s social wellbeing leading to feelings of low self-esteem and worthlessness as well as increased anxiety and decreased levels of happiness, resulting in depression^11,15–17^.

### Technology-based interventions to reduce social isolation and loneliness

Within the last decade several systematic reviews have focused on technology-based interventions for people who are experiencing or who are at risk of experiencing loneliness and social isolation^18–21^. Masi et al.^18^ in their meta-analysis, explored the efficacy of technology-based vs non-technology-based interventions across all population groups, notably, the mean size effect for technology-based interventions was − 1.04 (N = 6; 95% CI  − 1.68, − 0.40; *p* < 0.01), as opposed to − 0.21 (N = 12; 95% CI  − 0.43, 0.01; p = 0.05) for non-technology-based interventions. Choi et al.^19^ reported a significant pooled reduction in loneliness in older adults after implementing technology-based interventions (Z = 2.085, *p* = 0.037). Early technology-based interventions consisted of conference calls/video conferencing, text-based Inter Relay Chat and Emails^18–20^. Subsequent systematic reviews^21,22^ found that video conferencing was able to reduce loneliness in older particpants, however, this technology only helped to facilitate communication between existing, rather than new contacts. These types of intervention are therefore less beneficial for individuals who are socially isolated and struggling to establish connections with others.

During the Covid-19 lockdowns there was no possibility to provide or continue providing face-to-face individual or group interventions for lonely people. Moreover, even non-lonely people found themselves in situations where they could not maintain their social relationships through face-to-face interactions. Thus, the Department of Primary Care and Public Health in England recommended that avenues for mitigating feelings of loneliness should look to include web- and smartphone-based interventions^23^.

Virtual reality (VR) using a head mounted display (HMD) is considered qualitatively different from other technologies in that it has the ability to provide a sensation of immersiveness or ‘being there’^24^. VR technologies are becoming more accessible and comfortable with the creation of lighter more portable HMDs at a more affordable cost. This allows the technology to be used by a greater range of adults and members of vulnerable groups, e.g. adults with mobility impairments and older adults with age-related impairments. VR users, often represented as avatars, are able to meet and communicate in real-time with each other within a range of different scenarios. People are able to participate in social activities with new people, e.g. venturing off into new and exciting worlds (with nature scenes)^24^, travelling to different destinations around the world^25,26^ without leaving their homes and escaping their confined realties or engaging in horticultural therapeutic interactions^27^. Older adults are able to engage in social networking activities, including playing games with other people and attending family events through VR, users spoke very positively and expressed visible signs of enjoyment about their experience^28–30^. Virtual gaming is very popular among younger users with^31,32^ reporting that players experience significantly lower levels of loneliness and social anxiety when playing VR games compared within the real world.

Users taking part in VR interventions report being less socailly isolated, show less signs of depression, and demonstrate greater levels of overal well-being^24–27,33,34^. Widow(er)s in a VR support group showed a significant improvement during an 8-week intervention^35^. While both systematic reviews^33,34^ reported useful insights regarding the positive impact of VR technology on loneliness, most studies on VR environments included a small number of participants from specific populations, thus the reported findings have limited generalisability.

When VR is used as an intervention to reduce social and public speaking anxiety, it is found to be most effective as a mode of delivery for alternative therapeutic interventions such as Acceptance and Commitment Therapy^36^. Furthermore, Kim et al.^37^ found that patients with Social Anxiety Disorder (SAD) benefitted from the use of VR as an intervention, evidenced by short-term neuronal changes during exposure. They concluded that VR is useful as a first intervention for SAD patients who are unable to access formal treatment.

Various social VR platforms have emerged since 2013, e.g. VRChat, Altspace VR and RecRoom, however, the use of social VR as an intervention for reducing social isolation and loneliness is still a relatively new and unexplored field. Therefore, whilst there is research to support the effectiveness of VR as a tool to deliver therapeutic interventions and improve social well-being, there is limited research on the use of social VR as an online mechanism to decrease social isolation and improve group belonging.

### Innovation and contributions of this study

The current study is a cross-sectional study of the general population, socially isolated during the Covid-19 pandemic and who were using social VR platforms to interact with each other. This study addresses the limitations of previous studies, which have focused exclusively on specific groups within the population, i.e. older adults or VR gamers, or explored general well-being rather that loneliness and social anxiety. In previous studies the HMDs were often provided by the research team, meaning that there was a time restrain (frequency or length) in relation to the use of the VR technology by participants. This study is novel as it explores the effects of loneliness and social anxiety on a wider demographic of people, who have unrestricted access to HMDs and have been socialising in Altspace VR during the Covid-19 pandemic. This study is of an international character and utilises a mixed methods approach to explore the benefits of social VR to help reduce feelings of loneliness and social anxiety and to provide additional means by which social contact can be enhanced for vulnerable populations who may remain isolated post-pandemic.

### Research hypotheses

The following hypotheses were explored:

#### H1

Lower levels of loneliness and social anxiety are experienced when participants are in the social VR platform (online) compared with in the real-world condition (offline).

#### H2

Lower levels of loneliness and social anxiety are experienced by participants who are part of a group in social VR, i.e. members of a Virtual Social Group (VSG), than those who are not.

#### H3

Lower levels of loneliness and social anxiety are experienced by participants who have a group of friends in the social VR in comparison with those who do not.

#### H4

Lower levels of loneliness and social anxiety are experienced by participants who spend greater amounts of time in social VR.

## Methods

**Figure 1:**
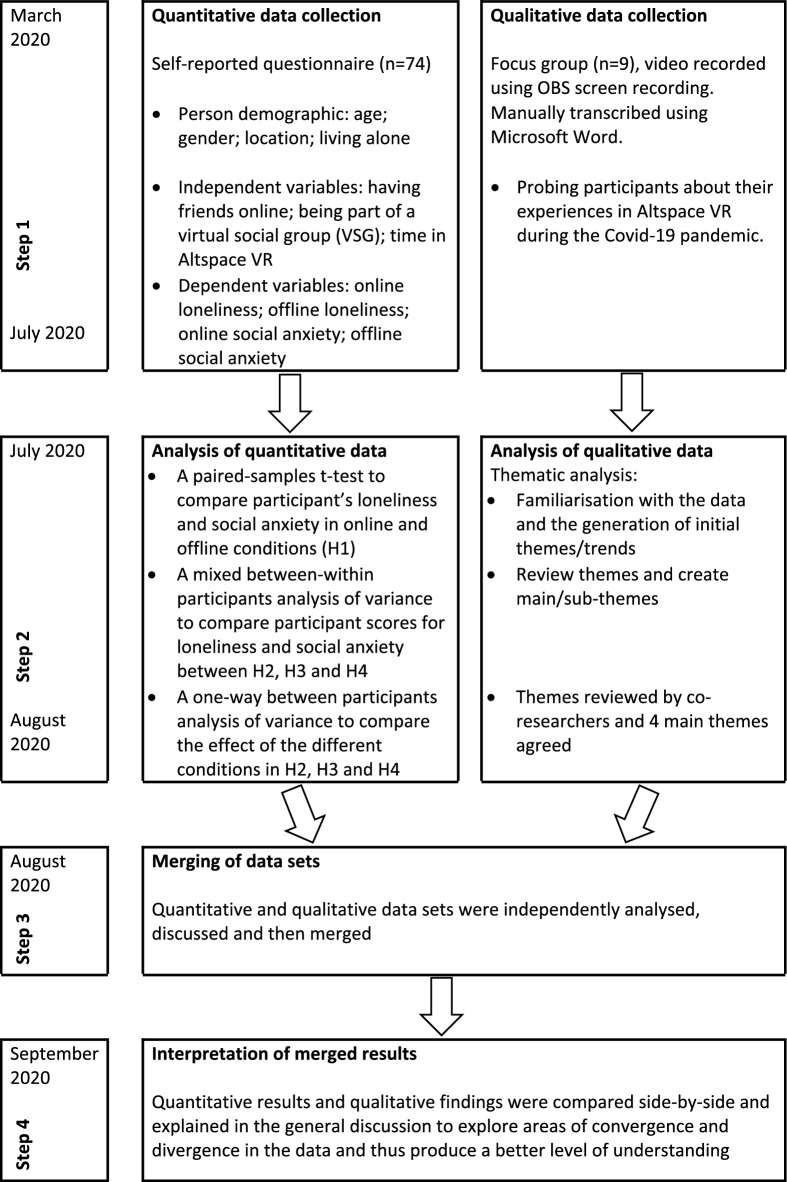
A convergent parallel mixed-methods model of the current research.

The study used a convergent parallel mixed-methods research design^38^ to collect both diverse quantitative and qualitative data (see Fig. 1). The study complied will relevant ethical regulations and was approved by the Research Ethics Committee of the University of Bolton, UK. Written informed consent was obtained from all participants.

### Collection of quantitative data

#### Participants

Participants were required to be English speaking, over the age of 18 and users of Altspace VR. A message of invitation was posted on different Discord community channels/message boards: Official Altspace VR; Educators In VR; Spatial Network; Humanism; Computer Science in VR; VR Church. 87 participants were recruited via an opportunity sampling method.

#### Materials and measures

A private research room was created inside Altspace VR to ensure that participants were able to complete the questionnaire undisturbed (see Fig. 5). The online questionnaire was created in Qualtrics XM and could be accessed across multiple devices: Oculus Quest, Oculus GO, Oculus Rift, HTC Vive and PC. The online questionnaire included sections about demographics, details of Altspace VR usage and sections assessing participant’s subjective feelings of loneliness and social anxiety. Measures of loneliness and social anxiety were collected for both conditions—real world (offline condition), followed by social VR (online condition).

The UCLA Loneliness Scale version 3^39^ was used to measure the subjective level of loneliness. This 20-item self-reporting questionnaire uses a four-point Likert scale, with 0 = “Never”, 1 = “Rarely”, 2 = “Sometimes”, 3 = “Often”. The loneliness score for each participant (range from 0 to 60) was determined as the sum of responses to all 20 items—higher scores reflecting greater loneliness. The UCLA Loneliness scale was adapted to include the word Altspace in the online condition as it was felt that this would further help participants to focus specifically on the online experience. No further adaptations were made to this questionnaire. The Social Phobia Inventory (SPIN) scale^40^ was used to measure the subjective level of social anxiety as it is effective in measuring the severity of social anxiety. This 17-item self-reporting questionnaire uses a five-point Likert scale, with 0 = “Not at all”, 1 = "A little”, 2 = “Somewhat”, 3 = “Very much”, 4 = “Extremely”. Adding the scores from each item produced a SPIN score for each participant. A higher SPIN score indicates more severe symptoms of social anxiety. No adaptations were made to the SPIN questionnaire.

#### Procedure

Participants who were interested in taking part in the survey were taken to the research room inside Altspace VR where they were sent a message with a link to the online questionnaire. Participants who clicked on the link were then presented with a browser window inside the room that only they could see. Participants who opened the questionnaire were first presented with the participant information sheet giving full details of the study. Information regarding withdrawal from the study and a list of additional support services were also provided in line with the University of Bolton’s ethical guidelines. After reading the study information sheet, participants were presented with the consent form for which full consent was required before they were able to move onto the survey.

The strategy for dealing with incomplete cases was to remove any participants who did not answer all of the questions, thus analysis was conducted on 74 participants. Exported data from the Qualtrics system was imported into the Statistical Package for Social Sciences (IBM SPSS, version 25). A Kolmogorov–Smirnov test (*p* > 0.5) was carried out to test for a normal distribution and histograms, nominal Q-Q plots and box plots were used to identify any outliers. Two outliers were found in the data for Social Anxiety in the offline condition and these were replaced with the mean of 17.54**.**

#### Characteristics of the sample

**Figure 2:**
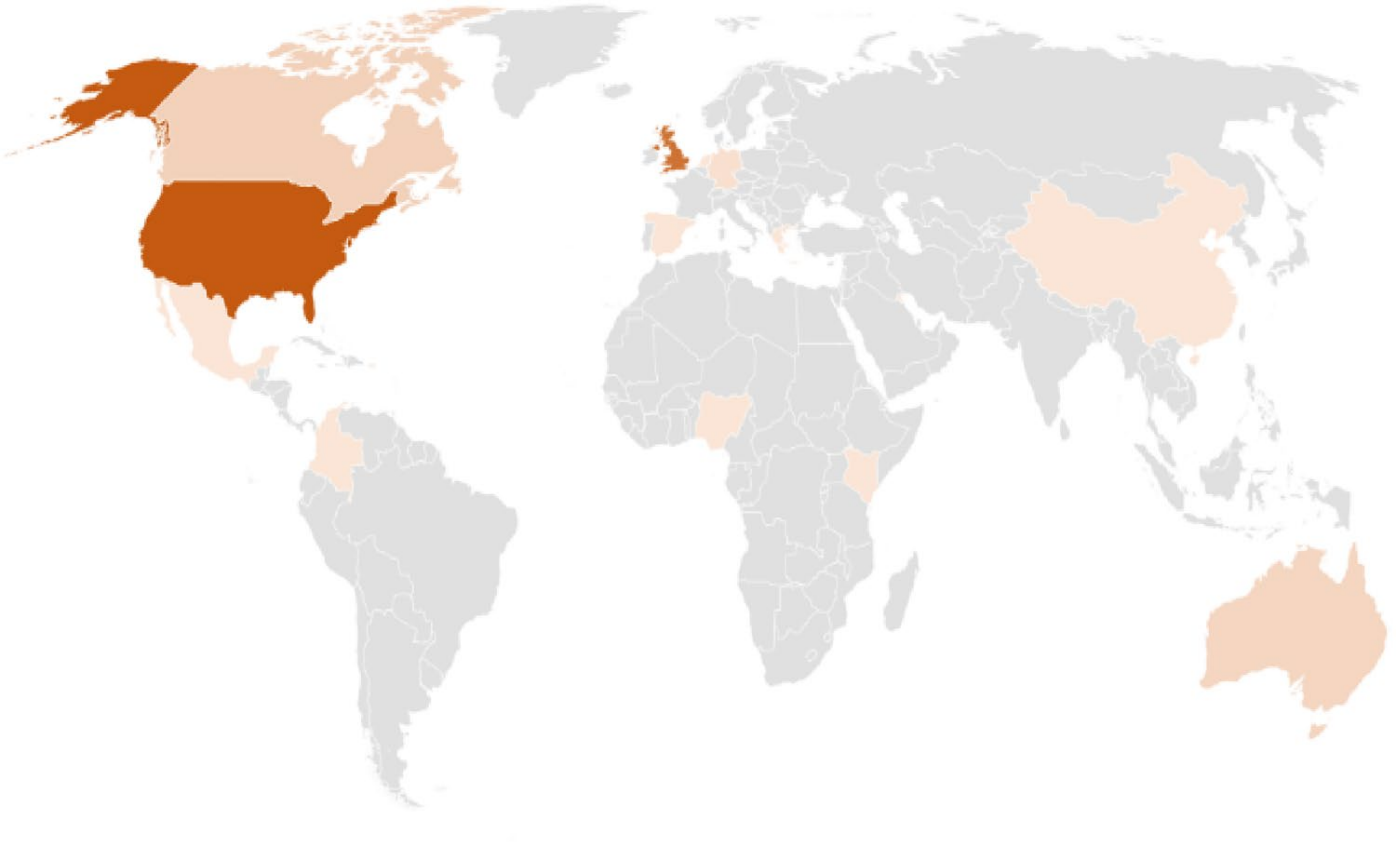
Participant’s location.

Of the total sample (n = 74), 46 were males and 28 females. The age range of respondents was 18–75 years (the split of valid participants is shown in Table 1). Participants were recruited globally (the geographical demographic is shown in Fig. 2). Out of these 74 participants, 31 participants (15 males, 16 females) were new to Altspace VR, having joined Altspace VR during the Covid-19 pandemic. 43 participants indicated that they had used Altspace VR before the outbreak of Covid-19.

**Table 1:** Age range of participants*.*

Age	Frequency	Percentage
18–25	17	23.0
26–35	14	18.9
36–45	13	17.6
46–55	17	23.0
56–65	10	13.5
66–75	3	4.1
Total	74	100.0

#### Change in loneliness and social anxiety

**Figure 3:**
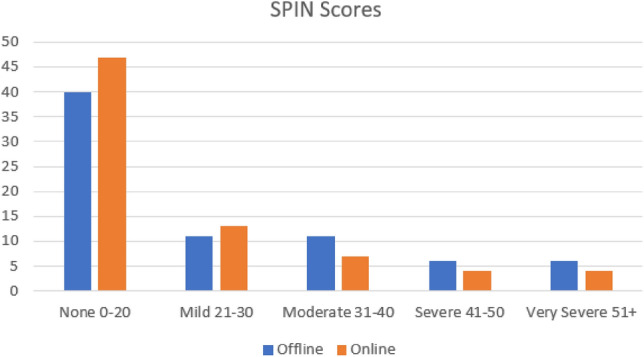
Participant’s SPIN Scores.

Figure 3 shows the breakdown of social anxiety scores in both the online and offline conditions. The data shows that the severity of social anxiety is higher in the offline condition, whereas participant’s levels of anxiety reduce when they are online.

The UCLA loneliness scale uses continuous scoring and so it is not possible to provide a similar breakdown for participant’s levels of loneliness. The effect that social VR has on the participant will be discussed in greater detail later.

**Figure 4:**
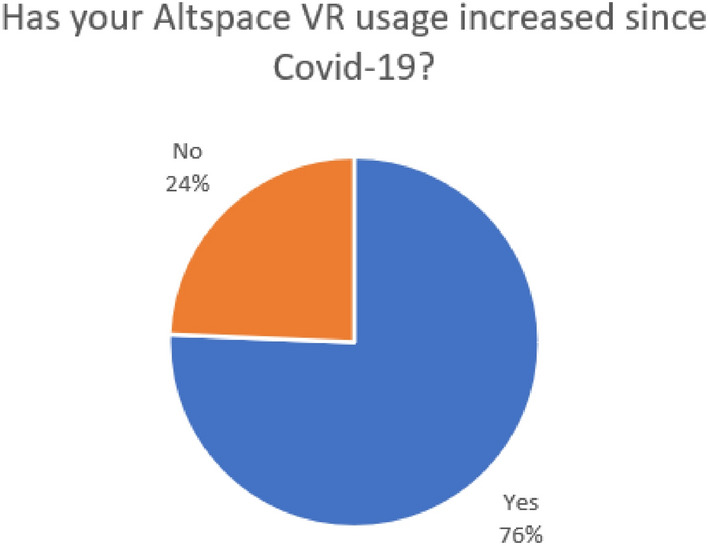
Participants usage of Altspace VR since Covid-19.

It was anticipated that during the Covid-19 pandemic and as a direct result of social distancing rules being imposed that general usage in Altspace VR would increase. Figure 4 shows that 76% of participants felt that their usage had increased and after calculating the average difference in usage (before and during Covid-19) an average increase per user of 11 h per week was reported.

##### *Hypothesis 1*

Hypothesis 1 predicted lower levels of loneliness and social anxiety are experienced when participants are in social VR (online) compared with in the real-world condition (offline) A paired-samples t-test was carried out to compare online (inside social VR) and offline (real-world) conditions for both loneliness and social anxiety. The results in Table 2 demonstrate a statistically significant decrease in the scores for loneliness from the offline condition (M = 20.53, SD = 14.80) to the online condition (M = 16.32, SD = 11.04), *t* = − 2.573, *p* < 0.05. A statistically significant decrease in social anxiety was found in the offline condition (M = 23.01, SD = 16.65) compared to the online condition (M = 16.34, SD = 13.09), *t* = − 5.80, *p* < 0.05. A small to moderate effect size^41^ was found for both variables (i.e. d loneliness = 0.32 and d social anxiety = 0.45).

**Table 2:** Results of t-test and descriptive statistics for loneliness and social anxiety in both online and offline conditions.

	N	M	SD	95% CI	t	df	D
Loneliness Online	74	16.32	11.04	[− 746, − 0.95]	− 2.573*	73	0.32
Loneliness Offline	74	20.53	14.80				
Social Anxiety Online	74	16.34	13.09	[− 8.97, − 4.38]	− 5.805*	73	0.45
Social Anxiety Offline	74	23.01	16.65				

*p < 0.05.

##### Hypotheses 2, 3 and 4

H2 predicted that lower levels of loneliness and social anxiety are experienced by participants who are part of a group in social VR than those who are not.

Being a member of a VSG means that the participant meets with a group or number of groups on a regular basis to take part in scheduled events, e.g. regular church services for members of VR Church; discussions around education each week for members of Educators in VR; mediation and relaxation sessions for members of the EvolVR group; and discussions on a whole range of matters relating to life in the Humanism group. 75.7% of participants (n = 56) indicated that they were a member of a VSG and 24.3% (n = 18) were not affiliated with any groups.

A one-way between participants ANOVA was carried out to compare the effect of being a member of a VSG separately for each of the dependent variables. No significant effect was found for loneliness in both the online condition F(1,72) = 0.17, *p* = 0.68 and offline condition F(1,72) = 1.63, *p* = 0.20. No significant effect was found for social anxiety in the online condition F(1,72) = 2.22, *p* = 0.14, however, a significant effect was found for social anxiety in the offline condition F(1,72) = 4.23, *p* < 0.05, η^2^ = 0.06 (a medium effect size). This finding suggests that participants who are part of a VSG experience less social anxiety (M = 20.80, SD = 15.64) than those who are not (M = 29.89, SD = 18.26) when in the real world (offline) condition.

H3 predicted that lower levels of loneliness and social anxiety are experienced by participants who have a group of friends in social VR in comparison with those who do not. This differs from Hypothesis 2 in that having friends in Altspace VR is seen as a deeper connection than simply taking part in group events where connections may not have been formed. Participants were grouped on whether they have a circle of friends in social VR with whom they regularly socialise with (52.7%, n = 39) and not (47.3%, n = 35).

A one-way between participants ANOVA was carried out to compare the effect of having a circle of friends separately for each of the dependent variables. A significant effect was found for loneliness in the online condition F(1,72) = 6.75, *p* < 0.05, η^2^ = 0.08 (a medium effect size), whereas no significant effect was found for loneliness in the offline condition F(1,72) = 0.03, *p* = 0.86. This suggests that participants who have a circle of online friends experience less loneliness (M = 13.28, SD = 11.02) than those who do not (M = 19.71, SD = 10.17). A significant effect was found for social anxiety in both the online condition F(1,72) = 6.82, *p* < 0.05, η^2^ = 0.09 (a medium effect size) and offline condition F(1,72) = 9.18, *p* < 0.01, η^2^ = 0.11 (a large effect size). This suggests that participants who have a circle of online friends experience less social anxiety (M = 12.72, SD = 12.64) than those who do not (M = 20.37, SD = 12.54) in both online and offline conditions.

H4 predicted that lower levels of loneliness and social anxiety are experienced by participants who spend greater amounts of time in social VR. There was a reasonable balance of participants who have been members of Altspace VR for more than 6 months prior to (n = 43) and who joined during (n = 31) the Covid-19 pandemic.

A one-way between participants ANOVA shows a significant effect for loneliness in the online condition F(1,72) = 4.68, *p* < 0.05, η^2^ = 0.06 (a medium effect size), whereas no significant effect was found for loneliness in the offline condition F(1,72) = 0.08, *p* = 0.93. This suggests that participants who have been members of Altspace VR for more than 6 months experienced less loneliness (M = 14.02, SD = 11.63) than those who joined during the Covid-19 pandemic (M = 19.52, SD = 09.43). No significant effect was found for social anxiety in the online condition F(1,72) = 2.13, *p* = 0.15, however, a significant effect was found for social anxiety in the offline condition F(1,72) = 4.77, *p* < 0.05, η^2^ = 0.06 (a medium effect size). This suggests that participants who have been members of Altspace VR for more than 6 months experienced less social anxiety (M = 19.51, SD = 16.82) than those who recently joined (M = 27.87, SD = 15.38).

## Discussion of quantitative results

Research into the use of web-based technologies and virtual worlds has consistently demonstrated positive effects of such interventions on an individual’s subjective feelings of loneliness and social anxiety. Hypothesis 1 of this study is therefore supported and is consistent with the earlier findings^31,32,42,43^ and a recent review^44^.

The results of this study in relation to hypothesis 2 were unable to support the assumption that being part of a VSG will reduce feelings of loneliness. The study was therefore unable to support findings from^32^ which reported that VR gamers who played as part of a guild were less likely to experience feelings of loneliness. Social identity theory^45^ provides a possible explanation for this. Teaming up with a specific VR gaming guild with the common purpose of defeating an enemy for example exerts a stronger sense of identity and group attachment compared to belonging to multiple virtual social groups, where an individual could have several social identities, thus group attachment is less salient. Furthermore, group attachment takes time to develop and within Altspace VR new VSGs are being created all the time. Future studies should look to explore the relationship between the membership duration and the strength of group attachment and the effect this has on subjective feelings of loneliness.

The results of this study support hypothesis 3 in that participants, who have a circle of friends with who they regularly socialise in social VR, experience lower levels of loneliness and social anxiety. This is consistent with the findings of^32^ who found that playing with known people helps to reduce feelings of loneliness and social anxiety. This also further supports the findings of^46^ who found that half of participants considered their gamer friends to be comparable to their real-life friends. As pointed out by^47^ in the Need to Belong Theory, people need frequent and meaningful interactions to feel fulfilled. The ability to form positive social interactions with people with which we feel most connected, i.e. a circle of friends that share our goals or with which we have a common purpose, promotes greater levels of satisfaction and generates greater feelings of belonginess, which in turn reduces our feelings of loneliness and social anxiety^48^.

The results of this study in relation to hypothesis 4 support the assumption that the longer a person has been in social VR the lower will be their feelings of loneliness. There was a significant reduction in feelings of loneliness in the online condition, but not in the offline condition. The explanation for the divergence is that both new and existing Altspace VR users were experiencing similarly high levels of loneliness in the real-world condition, due to the sudden enforced period of lockdown that was imposed upon them, and that whilst being in social VR for a longer period of time showed a greater reduction in feelings of loneliness, in the real world the length of time they had been using social VR was not significant. A possible explanation for this is that when returning to the real world a person is again faced with the challenges of the imposed social isolation and will therefore continue to experience greater levels of loneliness. The reverse situation was found for social anxiety with a significant reduction in social anxiety being found in the offline condition for participants who had been using social VR for longer. This is a useful finding because it shows that using social VR for longer periods of time can help to reduce feelings of social anxiety in the real world. As is suggested by^42^ social VR can be used to build up social capital and thereby help to improve a person’s social skills in the real world.

### Focus group

#### Participants

Nine participants (6 male, 3 female) who took part in the online questionnaire were later recruited to take part in a focus group. The demographics of this group are shown in Table 3. The focus group was made up of a wide mix of people from around the world. Participants were a mix of educators, students, developers and other professionals. Four of the participants were new to Altspace VR, having joined during the Covid-19 pandemic, whilst five had been in Altspace VR for more than 6 months. All the participants had previously attended at least one Educators in VR research event.

**Table 3:** Focus group demographics.

Participant	Gender	Age	Location
1	Male	50	Canada
2	Male	25	USA
3	Female	24	Netherlands
4	Male	53	Spain
5	Female	30	USA
6	Female	46	Greece
7	Male	36	Kenya
8	Male	23	Puerto Rico
9	Male	60	Canada

#### Procedure

**Figure 5:**
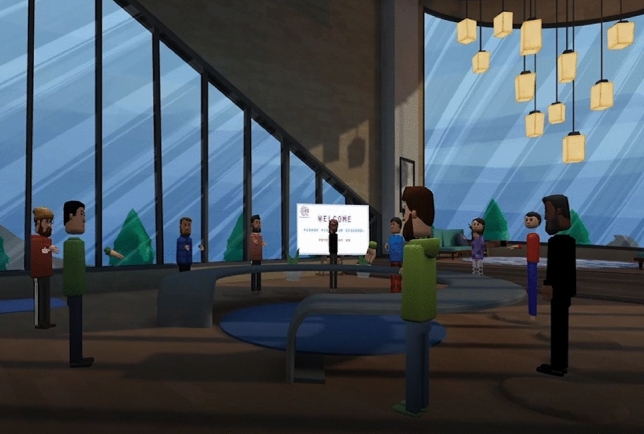
Virtual research room.

The focus group study took place in a private research room inside of Altspace VR (see Fig. 5), purposely created by the researcher. Only selected participants were able to join this room via a portal link provided by the researcher. The interview was recorded using OBS screen recording software on the researcher’s computer.

Prompts were kept to a minimum and questions were open-ended to elicit rich responses from participants. The focus group was later transcribed verbatim by the researcher. The transcript was analysed using a thematic data analysis approach as per the Braun and Clarke framework^49^. Thematic analysis is a suitable analytic approach to systematically establish patterns of meaning within qualitative data sets^50^. Microsoft Word was used to facilitate data management and the coding of themes. Participants’ responses were coded and themes identified.

## Qualitative results

Four superordinate themes with several subordinate themes were identified (see Table 4).

**Table 4:** Summary of superordinate and subordinate themes.

Superordinate theme		Subordinate theme	
1	Why the participant visits the social VR platform	i	Socialising in VR
		ii	Attending community events and learning new skills
		iii	Sharing ideas with professionals and like-minded people
2	How the participant sees their current situation	i	Introverted/anti-social in real life
		ii	Socially isolated owing to remote location and work/life balance
3	How the participant sees the social VR platform	i	Greater immersion/presence and therefore more like real life than other communication methods
		ii	More ways to connect and interact
4	How social VR is helping during the Covid-19 pandemic	i	Helps people feel less lonely
		ii	Helps to motivate and provide structure
		iii	Helps people to be less anti-social and reduce social anxiety
		iv	Helps to socialise with real life friends during lock-down

### Theme 1. Why the participant visits the social VR platform

Participants spoke freely about how they got involved in Altspace VR and what they believe to be the main reason they visit Altspace VR. Three sub-themes were discovered, although from the discussions it was clear that most, if not all, participants, valued the group interaction and attendance at events very highly.

(i) Socialising in VR

What was interesting about the group of participants in the focus group was that they were all connected due to their involvement with the Educators in VR community and not through friendship ties. Some participants highlighted that they initially joined Altspace VR to meet new people and then started building a network of professional relationships.

Participant quotes from the transcripts are given within the results section for each subordinate theme. For confidentiality purposes quotes from participants will be referenced as: Participant (P), followed by a number 1–9 and the participant’s gender M (male), F (female) e.g. “P1M”.

“In VR I hang out with friends and of course the [Educators in VR] research team, but I don’t hang out around the campfire as much anymore” (31-33,P3F).

The campfire in Altspace VR is a meeting place for new users to mingle, chat and make friends. New users to Altspace VR tend to levitate towards the campfire until they establish friendship groups and events in which to take part in. This participant has already established a network of meaningful friendships and they are now spending less unstructured time in social zones.

All participants highlighted that they had seen an increase in their usage during the Covid-19 pandemic. The imposed restrictions on physical meetups led to several participants using social VR to meet with real-world friends to satisfy their social needs.

“During this pandemic I have probably come in an hour or two more per day. Part of that was to connect with some of my friends. I got some friends to start coming into Altspace VR so we were able actually hang out in Altspace” (52-55,P5F).

“more recently, in the last month or so, because I work in the VR community and a lot of my personal friends have VR headsets, the people that I work with at the university, The people that are in my groups and in my sphere so to speak at the university are some of my best friends and so we have started having social meet-ups in VR for nothing other than social, like just for social meet-ups” (125-132,P1M)

(ii) Attending community events and learning new skills

All of the focus group participants recognised the value of taking part in regular events in social VR. In particular, participants were positive about the opportunities that exists within Altspace VR to collaborate with others to expand and learn new skills. Community involvement within Altspace VR generates a strong sense of belonging thus reducing feelings of loneliness and social anxiety.

“I got inspired by the Covid situation to host events, so it inspired me to bring people together. I think if the Covid situation did not happen I wouldn’t have organised these research meetings to be honest, so it was pretty much the catalyst to hosting events” (161-165,P3F)

“One thing I love about the Altspace environment is the Educators forum because I have joined philosophy classes, I’ve done Psychology classes, I’ve really interacted. In fact, I started a talk show, [ ] my own event, and that’s one thing that I love about Altspace, so I do love this place” (72-78,P7M)

(iii) Sharing ideas with professionals and like-minded people

Altspace VR allows users to create their own events and to share knowledge with other users. There are a wide range of different interest groups within Altspace VR. Establishing common interests with others is a cornerstone to forming positive and meaningful relationships. Establishing a network of contacts is also beneficial by encouraging, giving advice and supporting each other in difficult times^51^. Several of the participants commented that social VR is a useful tool not least during periods of enforced social isolation, but also to those who find themselves unable to form such relationships within their existing real-world social networks.

“I entered Altspace mainly for the Educators in VR conference and after that, during the Covid crisis obviously I stayed because it is a perfect place to find people that have a similar interest with mine” (62-64,P6F). “It’s almost impossible where I live to find people with similar interests like mine, so this is probably the only way for me to find people with similar interests” (188-190,P6F)

“I love coming here because there are so many truly brilliant people with so much to learn and so many interesting things to hear and see” (105-107,P9M)

### Theme 2. How the participant sees their current situation

Although participants were not specifically asked, they took it upon themselves to reflect how they see the current situation and their specific circumstance in terms of being socially isolated. Participants felt that they were socially isolated and less social for several reasons. These have been broken down into the following sub-themes.

(i) Introverted/anti-social

Several participants stated that they are socially inhibited and anxious individuals, who find socialising in the real world more challenging, whereas social VR offers a less intimidating way for them to meet and make friends.

“If you struggle with social interaction, VR is a little less intimidating, I would say. I really think these platforms are a great way to connect and less intimidating as well” (240-245,P3F)

“Prior to Covid I was actually pretty like unsocial, I still kind of am unsocial, but it seems as though now society is kind of like bending towards introverts so in a sense it’s like the market’s benefiting my type so like in a sense I’m becoming increasingly more social” (18-22,P2M).

(ii) Socially isolated due to remote location and work/life balance

Some participants lamented that their geographic location or work/life balance in the real world made it very difficult for them to meet and to have frequent interactions with people with similar interests to theirs. This aspect makes them at a greater risk of loneliness to others. Social interaction within social VR is not restricted by geographic location and so these participants feel that this has helped to enhance their social interaction with others.

“I use VR to socialise because I live in a little village so for me it’s the only way to meet people, to communicate with people etc because normally I don’t meet people in the real life. With my friends and with my brother etc so I use the VR to socialise okay” (40-43,P4M)

“I went on sabbatical in September this academic year I spent my entire summer, last year outside hiking and camping and all of that and then all of a sudden I was inside doing research and I was isolated from my community. I feel like my work community is my community, you know, and I felt like I lost my community and I felt like I found a new one in Altspace” (259-265,P1M)

### Theme 3. How the participant sees the social VR platform

Several participants elaborated in detail on how they felt that social VR helped them to connect with people in ways that were better than alternative digital communication methods such as video conferencing, text chat or social media.

(i) Greater immersion/presence

Immersion and presence are important characteristics within VR because the aim after all is to replicate, to some degree, the feelings of being within the real world. The more this is made possible the more useful VR will be in combating feelings of loneliness and social anxiety during periods of prolonged isolation in the real world.

“I’ve been in here with students for tutorials and […] students have said that they feel more presence with other students in this environment” (108-111,P9M)

“I’m a perceptual psychologist so I even think about it from the view of like it feels like some of the spaces that I go into now in Altspace really regularly feel in my head like real spaces that I go to so when I feel like I go to a couple of events in the afternoon in Altspace and then I take the headset off it kind of feels like I left my house and I went out and did something and then came back, it doesn’t feel like I was in my house the whole time” (154-160,P1M)

(ii) More ways to connect

In addition to the greater immersion and presence that VR can create, Altspace VR also gives individuals the ability to control and create their own environments for social interaction. It is not possible within the real world for most of us to simply create our own hang-outs or to control our environments so easily. This allows people to therefore interact in ways that up until now have not been possible. Several participants linked the ability to create stimulating and exciting environments in the Altspace VR to something that they can feel proud of, and this gives them social capital over other users with less advanced skills in world creation. This in turn helps to improve their ability to socialise and build further friendships in social VR that they would not have been able to build in the real world.

“I made a beach environment, a beach world and there are other ones out there, but I made a custom private one for me and my friends to meet in and so we meet in there and other places and we bounce around and look at different places but we often find somewhere like a private room where we can actually have a nice private conversation and we don’t have to worry about anyone interfering and everyone said its fantastic it really allows us to connect in ways, you know like those personal chats you have with close friends that it’s hard to do in any other medium, it feels a little more natural in VR to do that and so it’s been fantastic, we’ve been really enjoying it” (132-142,P1M)

“Since coming in here now [my friends] are like world building and have created some really awesome spaces in here and so we go in and check out the space that they just created and so I’m still kind of doing project oriented hang-outs as far as like we will be like oh that lighting needs to be a little different and stuff like that but it’s been a really fun way to hang out with people that I already may have been friends with before all this happened but now that this happened they are starting to come into this space so we can connect even more often” (214-222,P5F)

### Theme 4. How social VR is helping during the Covid-19 pandemic

In the second part of the focus group, participants were asked to think about how they thought Altspace VR was helping them specifically during the Covid-19 pandemic and whether they thought that others could benefit from this experience too. The responses were very positive and provided a great deal of insight into how Altspace VR is helping them to deal with loneliness and social anxiety during Covid-19. A number of key sub-themes emerged from this category.

(i) Helps people feel less lonely

Several participants said that social VR helps them to feel connected with a circle of friends and that this helps to reduce feelings of loneliness and depression.

“I feel it really does help me in social isolation. I have been on sabbatical this last year so my whole year has been about isolation even before Covid-19, I’ve been working a lot on my own and that sort of thing so yeah becoming part of the community in Altspace, collectively in the different ways that I have has had a huge impact on my mental health. I was getting a little depressed in the fall and having this community has really felt like that it brought me out of it a bit” (147-154,P1M)

“By the second semester I only had like one course and we were like really concentrating on a specific project and everything and it was like really limiting me to go outside and do some other stuff. Even though I’m an introvert but I do feel like I really wanted to go outside and have some fun. I really like to see other stuff around me and doing all this stuff here in VR kept me really engaged with the communities” (191-197,P8M)

(ii) Helps to motivate and provide structure

Having a purpose and being occupied with an interesting project and subsequently conversing about its progress/issues with others in social VR were perceived as motivational factors, which helped them to deal with the imposed social isolation.

“Events really motivated me to keep busy also when I was in social isolation for two months. Yeah, two months is a long time you know to not get out of your house so that was great I created some sense of purpose and it was really heart-warming to see everybody come together and really interesting people as well. Everybody has something cool to share and was very helpful so that gave me some energy, you know to just keep on going and make the best out of the situation” (166-173,P3F)

“I finally have a structure for a project that I have been thinking about for over a year now and

having these interactions in here and talking to people allowed me to bring a clear picture of how I can start a project I have been thinking about and start building it inside Altspace, so that’s a big plus for me” (178-182,P6F)

(iii) Helps people to be less anti-social and reduced social anxiety

Several participants explained that social VR is “a great way to connect and less intimidating as well” for socially anxious, i.e. “unsocial” and “introverted” people, who as a result often feel lonely. In addition, social VR is a convenient tool for social interactions as it brings people closer “especially during these situations, but not only during like pandemics”. (240–243,P3F)

“In my case the Covid increased my social interaction with people because I’m a pretty anti-social person in real life so for me this has increased ten-fold my social interaction in general” (174-176,P6F). “Covid pushed people inside spaces like VR and made my social interactions far easier to have” (186-188,P6F). “I am in sort of a group, let’s say of people who have problems with connecting with people, this is awesome. This is definitely a big plus and I would like more of this” (322-324,P6F)

“I was, I guess, somewhat socially isolated before coming in Altspace I tend to just like to work on projects and stay at home or be at work, but since coming in Altspace I’ve definitely started experiencing more of the social aspect of living like making connections with other people in ways that aren’t strictly like a project that I’m working on and so that’s been nice” (202-208,P5F). “I do think that VR can help us, those of us who are socially isolated or have social anxieties of some sort. It does make it more accessible for us to be able to go into a space and interact with people. For instance in real life, if you were to have social anxiety and you start feeling almost like a panic attack coming on, that would prevent you from going into a real life space, whereas in VR you […] can say, oh I have to go really easily and you’re back in your home and you can work through whatever may have come up with social anxiety. So I do think it makes social interactions more accessible in those cases” (307-316,P5F)

(iv) Helps to socialise with real life friends during lock-down

Another idea that surfaced among the participants is the potential to use social VR as a mode of interaction/engagement with real-life friends/family members who live afar. Participants expressed the view that the current restriction on face-to-face contact could to some extent be counterbalanced by inviting real-world friends into social VR to socialise.

“The fully social part of VR has happened because of the Covid-19 situation, because I used to go for dinners with people like every month, […] and we can’t do the real world social, so we are trying to do the VR social” (142-146,P1M)

“Once everyone went into social isolation for Covid I actually started hanging out with a friend that lives 3 hours away from me more than before because before it would be a 3 hour drive, but then once all this happened, I actually convinced them to come into Altspace” (208-212,P5F) “It’s been a really fun way to hang out with people that I already may have been friends with before all this happened but now that this happened they are starting to come into this space so we can connect even more often. (218-222,P5F).

## Discussion of qualitative findings

Overall, participants’ commentaries to Theme 1 reconfirm that their usage of social VR has increased during the period of imposed social isolation and restrictions on physical meetups due to the Covid-19 pandemic. They were using social VR to meet with real-world friends to satisfy their social needs and continue to receive support from people they are close to; or to mix socially with other users who they meet either at a “campfire” or whilst taking part in regular events inside of the social VR platform, thus expanding their social network of non-intimate contacts. As a result, they felt less lonely online (whilst being in Altspace VR) as they felt like they were in the same space together. Interestingly, participants noted that they also benefited emotionally from meeting like-minded people/professionals and sharing ideas with them, getting support and advice, and working together in real-time. This is a new explanation why people use VR technology, which did not surface in the earlier research studies. Nonetheless this reason ties with the Need to Belong Theory^47^. This is useful to help us to understand why users visit Altspace VR in general and during the enforced social isolation period.

**Figure 6:**
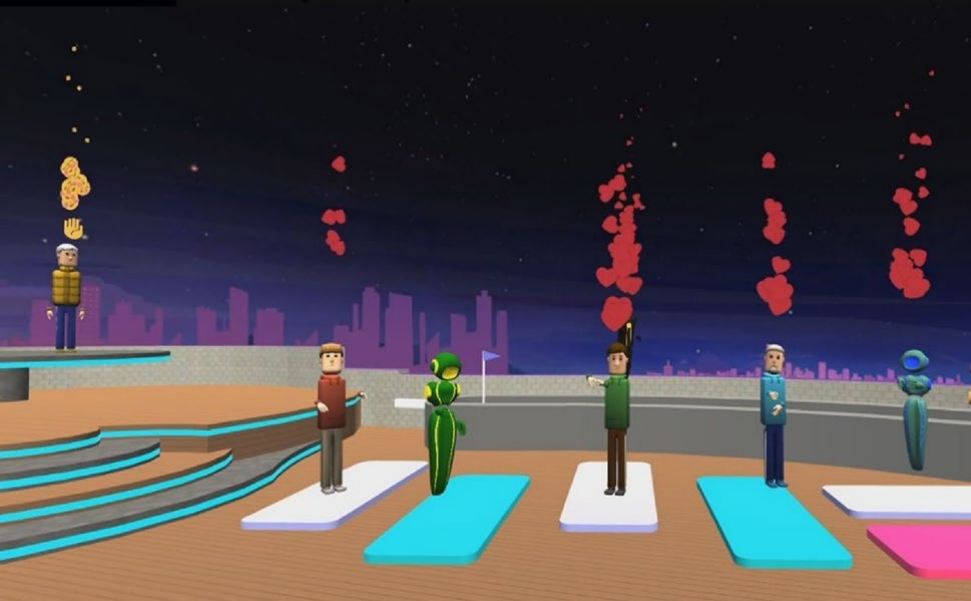
Use of emojis to communicate in Altspace VR.

In theme 2 participants’ responses reiterate what has already been explained in the literature that shy, socially inhibited and anxious individuals find online anonymity liberating and less inhibited than the real world^52^. Moreover, in Altspace VR it is also possible to make use of non-verbal communication such as emojis or emoticons (see Fig. 6).

Some participants commented that their geographic location or work/life balance in the real world made it very difficult for them to meet people with similar interests. The social internet, e.g. Facebook^53^ and video conferencing^54^ have long been used to socialise with friends and family and have been found to be an affective intervention for reducing loneliness. Theme 3 considers that social VR could be regarded as the latest endeavour within this field as individuals are able to create their own exciting hangouts, e.g. a beach or a city from Ancient Greece. Furthermore users are able to easily control environments and restrict entry. This allows people to interact in ways that up until now have not been possible.

Findings in Theme 4 give a clear indication that social VR helps to reduce feelings of loneliness, and this further supports the findings of^32^. Social interactions in social VR are also particularly attractive to those who are lonely or shy/socially anxious/self-conscious or have poor social skills, etc. as they feel more in control of their online interactions and feel that they have a broader range of topics that they are able to discuss compared with in the real world^55^. Lonelier people also feel that they can be more themselves in online social interactions than in the real world^56^.

## General discussion

People use social VR for many different reasons: to socialise with new and existing friends; to join social interest groups; to learn new skills and generally to be part of a larger community of people (including other professionals) than those that they are part of in the real world. Social VR attracts a wide range of people because of the ease in which people can meet people with similar interests to their own, although it could be argued that up until the recent Covid-19 pandemic social VR tended to attract a greater amount of people who found real-life social interaction difficult. The results of this study show a reduction in social anxiety in individuals with moderate, severe and very severe social anxiety in the online condition, i.e. when using social VR. The increase in availability of VR headsets in recent years has led to an expansion in usage of social VR and the recent Covid-19 pandemic and subsequent social distancing rules led to more people and organisations making a greater use of VR to communicate and carry out their daily business and routines during the prolonged period of social isolation. Social VR also enables people to collaborate in ways not possible within the real world, reducing geographic restrictions and breaking through communication barriers by using visually stimulating content creation tools to enhance the process of human interaction through world-building and event hosting.

The main objective of this study was to explore whether social VR could be used to help reduce feelings of loneliness and social anxiety amongst people confined to their homes and away from their regular friendship groups and social connections, i.e. when the quantity and quality of their social network is gravely affected. Overall, the synthesised results of the present study show that participants experience a statistically significant reduction in loneliness and social anxiety when in social VR than in the real world during prolonged periods of imposed social isolation. Qualitative findings support/validate the quantitative results for H1. Thus, the evidence shows that social VR can decrease the sense of loneliness and social anxiety with users and have an overall positive effect on their emotional and social wellbeing.

The qualitative data diverges from the quantitative results presented for H2 that addressed the effect of being part of a VSG separately for loneliness and social anxiety. The quantitative results showed no significant effect for loneliness in the online and the offline conditions, whereas participants’ views showed that being a member of a VSG created a sense of belongingness and helped them to feel less lonely and depressed. Quantitative data showed no significant effect for social anxiety when an individual is a member of a VSG or not; but revealed a medium effect for social anxiety in the offline condition indicating that users, who are part of a VSG and subsequently take part in regular group events, experience less social anxiety in real world (i.e. offline), than those who are not part of a VSG. Participants who are part of a VSG were positive about the possibilities of social VR and being part of a VSG, because this setup helped shy and socially inhibited individuals to observe conversations, use emojis to show emotions rather than speak, use the online anonymity to get over the discomfort of social interactions and gradually become more connected and accepted by other members of the VSG. This prepares socially anxious individuals to handle being out there (in online and the real world).

Qualitative findings are in line with the quantitative results for H3 in that the degree of loneliness and social anxiety is also further reduced by factors such as having a circle of online friends. Social VR allows people to meet others who share similar interests, this is more difficult within the real world for people who struggle with social anxiety or who live in remote locations for example, or as was the case with this study, people who were confined to their homes due to social distancing rules during a pandemic. The qualitative data helps to produce a better understanding in relation to ‘online friends’ as these include individuals who were met in social VR and real-life friends who currently live afar and were invited to join the social VR platform.

The qualitative findings somewhat converge with quantitative results for H4 in that online loneliness reduces with the length of time the participant has been using social VR, i.e. participants who had been using social VR for greater than 6 months experienced less loneliness than those who joined during the Covid-19 pandemic. The length of time the participant had been using social VR had no effect on their feelings of loneliness in the real world. Comments from participants who have been members of Altspace VR for more than 6 months revealed that finding a new (online) community that supports their need to belong and provides meaningful and positive social interactions acted as an antidote to the loneliness that they experience in the real world. Individuals who struggle to build meaningful relationships in the real world due to social anxiety and other social phobias turn to social VR as it provides a less confrontational way in which to form and maintain social relationships with others and therefore help to reduce feelings of loneliness and social anxiety.

## Research limitations and implications

The heterogeneity of the sample for the quantitative survey enabled conclusions to be drawn regarding the participant experience in Altspace VR, their subjective feelings of loneliness and social during the Covid-19 pandemic. However, in interpreting the views of participants in the focus group it should be stressed that the sample of participants was solely recruited from the Educators in VR research event and that this may not represent the views of others who do not take part in such events. Although the reported themes were clearly identified, there remains a possibility that additional themes would be detected should the views of participants from a wider pool be collected.

It is the researcher’s understanding that this is the first study that has exclusively focused on participant’s feelings of loneliness and social anxiety during a period of enforced prolonged isolation whereby social VR has been utilized as an intervention to help reduce such feelings. The results offered here, should therefore be taken as a starting point upon which further empirical studies could be built. Longitudinal investigations could be carried out to further assess the suitability of social VR as an intervention to help reduce loneliness and social anxiety amongst specific communities, e.g. remote learners/workers, people living alone or in care, the less physically able, prisoners and other sub-groups of people facing loneliness and social anxiety whereby their ability to socialise with other is in some way restricted. Future research would also need to provide accurate estimates of the prevalence of loneliness and social anxiety in these sub-groups.

## Conclusion

The COVID-19 pandemic forced people to change the way in which they connected with others during lockdown. Social VR helped to improve social connectedness during the COVID-19 pandemic and reduce “lockdown loneliness”. Post-pandemic it is necessary to recognise the additional needs that face society, especially vulnerable people and those struggling with mental health issues resulting from lockdown. Social VR can, therefore, be a way of further supporting people facing social isolation, loneliness and social anxiety. Social VR platforms may be virtual, but the relationships we build in them are very real.

## Data Availability

All data generated or analysed during this study are included in this published article or in the accompanying Supplementary Information file.
